# 568. Nutrition Interventions in Older Adult Burn Patients

**DOI:** 10.1093/jbcr/irag033.308

**Published:** 2026-04-06

**Authors:** Ashlyn Elftmann, Marah Kays, Alexandra M Lacey, Nicholas Larson, Kathleen S Romanowski, Colette Galet, Jessica Reynolds, Dhaval Bhavsar, Lauren B Nosanov, Theresa L Chin, Hannah M Hamm, Mark J Johnston

**Affiliations:** University of Minnesota, Hopkins, MN; Regions Hospital, Minneapolis, MN; Regions Hospital, Saint Paul, MN; Regions Hospital Burn Center, Minneapolis, MN; Shriners Children's - Northern California, Sacramento, CA; University of Iowa, Iowa City, Iowa; The University of Kansas Medical Center, Kansas City, KS; The University of Kansas Health System, Kansas City, KS; Emory University School of Medicine, Atlanta, GA; University of California Irvine, Orange, CA; Hamilton Health Sciences, Hamilton, ON; Regions Hospital, Saint Paul, MN

## Abstract

**Introduction:**

Older adults are known to have a high risk of malnutrition at presentation, which is associated with increased morbidity and mortality. This study focuses on nutritional interventions and outcomes in older adult burn patients.

**Methods:**

We utilized a multi-center database that specifically assessed the outcomes of older adult burn patients (60 years+). Patient demographics, hospitalization data, and clinical course were analyzed using a post hoc pairwise test with Bonferroni correction. A mixed effect generalized linear model was run specific to survival as an outcome measure. Patients who elected to go comfort care were excluded.

**Results:**

The study group included 712 patients, nourished (admission albumin ≥3.0 g/dL, n = 442) and malnourished (admission albumin <3.0 g/dL, n = 270) with 57.9% of nourished and 73.7% of malnourished patients receiving supplemental nutrition. The malnourished group presented with increased total body surface area burns (*p* = < 0.001), higher revised Baux scores (*p* = < 0.001), and higher frailty score (*p* = < 0.001). The malnourished group had a statistically significant difference in need for supplemental nutrition (*p*=.001). However, the type of supplemental nutrition was not significant between groups. Mortality (*p* = < 0.001) showed the only statistically significant difference. When stratified by admission albumin, ARDS (*p*=.001) and catheter-associated blood stress infections (*p*=.001) were seen at higher rates in those with lower albumin levels. When comparing survivors to non-survivors, the differences between TBSA (*p* = < 0.001), modified Baux score (*p* = < 0.001), admission albumin (*p* = < 0.001), and length of ICU stay (*p* = < 0.001) were statistically significant. Non-survivors had higher rates of supplemental nutrition (81.8%), specifically in the form of tube feeds (93.7%) (*p* = < 0.001). Non-survivors had higher rates of ARDS (16.9%, *p* = < 0.001), cardiac arrest (13%, *p* = < 0.001) and abdominal compartment syndrome (7.8% *p* = < 0.001). A mixed effect generalized linear model for mortality revealed that for every one-point increase in admission albumin, the odds of death lowered by 54%.

**Conclusions:**

This retrospective study demonstrates those older adults who presented with malnourishment had higher TBSA burns and increased rates of complications. Those burn patients who survived had higher rates of nutritional support than non-survivors with non-survivors. Admission albumin correlated strongly with risk of mortality should be utilized as an additional marker to help predict risk mortality in this population.

**Applicability of Research to Practice:**

Our findings suggest that nutritional status prior to burn injury and in the post burn resuscitation is vital to improving survival in older adult patients.

**Funding for the study:**

N/A.

